# 'ZP domain' of human zona pellucida glycoprotein-1 binds to human spermatozoa and induces acrosomal exocytosis

**DOI:** 10.1186/1477-7827-8-110

**Published:** 2010-09-11

**Authors:** Anasua Ganguly, Pankaj Bansal, Tripti Gupta, Satish K Gupta

**Affiliations:** 1 Reproductive Cell Biology Laboratory, National Institute of Immunology, Aruna Asaf Ali Marg, New Delhi-110 067, India

## Abstract

**Background:**

The human egg coat, zona pellucida (ZP), is composed of four glycoproteins designated as zona pellucida glycoprotein-1 (ZP1), -2 (ZP2), -3 (ZP3) and -4 (ZP4) respectively. The zona proteins possess the archetypal 'ZP domain', a signature domain comprised of approximately 260 amino acid (aa) residues. In the present manuscript, attempts have been made to delineate the functional significance of the 'ZP domain' module of human ZP1, corresponding to 273-551 aa fragment of human ZP1.

**Methods:**

Baculovirus-expressed, nickel-nitrilotriacetic acid affinity chromatography purified 'ZP domain' of human ZP1 was employed to assess its capability to bind and subsequently induce acrosomal exocytosis in capacitated human spermatozoa using tetramethyl rhodamine isothiocyanate conjugated Pisum sativum Agglutinin in absence or presence of various pharmacological inhibitors. Binding characteristics of ZP1 'ZP domain' were assessed employing fluorescein isothiocyanate (FITC) labelled recombinant protein.

**Results:**

SDS-PAGE and immunoblot characterization of the purified recombinant protein (both from cell lysate as well as culture supernatant) revealed a doublet ranging from ~35-40 kDa. FITC- labelled 'ZP domain' of ZP1 binds primarily to the acrosomal cap of the capacitated human spermatozoa. A dose dependent increase in acrosomal exocytosis was observed when capacitated sperm were incubated with recombinant 'ZP domain' of human ZP1. The acrosome reaction mediated by recombinant protein was independent of Gi protein-coupled receptor pathway, required extra cellular calcium and involved both T- and L-type voltage operated calcium channels.

**Conclusions:**

Results described in the present study suggest that the 'ZP domain' module of human ZP1 has functional activity and may have a role during fertilization in humans.

## Background

Mammalian oocyte is surrounded by a glycoproteinaceous extracellular coat termed as zona pellucida (ZP). During fertilization, the ZP matrix plays a crucial role by serving as a substrate for sperm binding, as well as an agonist for regulated exocytosis of the spermatozoon's acrosomal vesicle and facilitates avoidance of polyspermy [1]. It also acts as a protective barrier around the embryo during early stages of its development till the implantation of the blastocyst in the endometrium takes place. Human ZP matrix is composed of 4 glycoproteins designated as zona pellucida glycoprotein-1 [ZP1; 638 amino acid (aa)], -2 (ZP2; 745 aa), -3 (ZP3; 424 aa) and -4 (ZP4; 540 aa) [2-4]. The role of respective ZP glycoproteins during different stages of fertilization has been a subject of intense scrutiny. Studies employing recombinant human ZP3, expressed in various expression systems, suggest that as in mouse, in humans, ZP3 also binds to the capacitated spermatozoa and induces acrosomal exocytosis [5-12]. The role of human ZP3 as putative primary sperm receptor has been further confirmed by employing immunoaffinity purified native ZP3 from human oocytes [13,14]. In contrast to mouse model, in humans, ZP4 [pseudogene in mice, 15] also binds to the anterior head of the capacitated acrosome-intact spermatozoa and induces acrosomal exocytosis [9,11-14]. Recent studies from our group employing baculovirus-expressed recombinant human ZP1 have demonstrated its role in binding to the human sperm and induction of acrosome reaction [16], whereas in murine model, ZP1 has been postulated to cross-link the filaments formed by ZP2-ZP3 heterodimers [17] and may not have any direct role in induction of acrosome reaction [18]. Similar to murine model, in humans, ZP2 fails to induce acrosomal exocytosis in capacitated human spermatozoa and predominantly binds to acrosome-reacted spermatozoa thus, acting as a secondary sperm receptor [1,9,11-14].

The biochemical characterization of ZP glycoproteins revealed that these share several common structural features that include i) N-terminal hydrophobic signal peptide sequence, ii) potential N- and O-linked glycosylation sites, iii) a C-terminal hydrophobic transmembrane-like domain (TMD), iv) a potential consensus proprotein convertase (furin) cleavage site (CFCS) upstream of TMD, and v) 'ZP domain' [19-21]. The formation of ZP matrix involves regulated proteolysis at CFCS by a member of the furin convertase family [22]. The 'ZP domain' consists of approximately 260 aa including 8 conserved Cys residues and is predicted to have high β-strand content with additional conservation of hydrophobicity, polarity and turn forming tendency at a number of positions [21]. 'ZP domain' has been shown to play an important role in polymerization of extracellular matrix proteins including ZP matrix [20,23]. This domain is also found in other proteins like the transforming growth factor (TGF)-βR III, uromodulin, pancreatic secretory granule protein GP2, α- and β-tectorins, DMBT-1 (deleted in malignant brain tumor-1), NompA (no-mechanoreceptor-potential-A), Dumpy and Cuticulin-1, *Drosophila *genes *miniature *and *dusky*, etc. [20,21]. 'ZP domain' has a bipartite structure with ZP-N and ZP-C sub-domains separated by a linker region [21]. The ZP-N sub-domain has been shown to be self-sufficient for polymerization [23]. Recently, 2.3Å crystal structure of the ZP-N sub-domain of murine ZP3 has been solved which will provide important framework to study the 'ZP domain' family proteins [24]. The role of ZP-C sub-domain is not as yet clearly defined.

In this manuscript, the functional significance of 'ZP domain' of human ZP1 has been investigated. The human *Zp1 *gene encodes a 638 aa long protein, which has a 25 aa long N-terminal signal peptide, a 'ZP domain' ranging from 279-548 aa and a tetrabasic CFCS, RQRR (552-555 aa) upstream of TMD. Human ZP1 'ZP domain' (273-551 aa residues; ZP1_273-551aa_) has been cloned and expressed using baculovirus expression system to obtain it in the glycosylated form. Recombinant ZP1_273-551aa _has been investigated for its binding to capacitated human spermatozoa, induction of acrosomal exocytosis and delineation of the downstream signalling events associated with acrosomal exocytosis using pharmacological inhibitors.

## Methods

### Expression of 'ZP domain' of human ZP1 using baculovirus expression system

To clone and express ZP1_273-551aa _in baculovirus, human ZP1 fragment corresponding to nucleotide (nt) 757-1653 (897 bp) was used as template which was PCR amplified from a commercially obtained adaptor-based human ovarian cDNA library (Marathon Ready cDNA library, BD Biosciences, Clontech, USA). Primers were designed based on the sequence published in the GenBank having the accession number NM_207341. The sequence for a 6× His tag was inserted in the reverse primer to facilitate easy purification of the expressed protein employing Ni-NTA affinity chromatography. The PCR amplification was performed in 50 μl reaction volume comprising of a 10× high fidelity PCR reaction buffer [600 mM Tris sulfate (pH 8.9), 180 mM ammonium sulfate], using 10-20 ng of the template DNA, 50 pmole of the forward (5'-CGGGATCCCGGCAACACAGCTACTGTC-3') and the reverse (5'-CGGAATTCTTA GTGGTGGTGGTGGTGGTGTGTAGTGCCAGTGCTACA-3) primers, 50 mM MgSO_4, _10 mM dNTPs and 1 unit (U) of Platinum Taq DNA polymerase high fidelity (Invitrogen Corp., Carlsbad, CA, USA) for extension. The template was initially denatured at 94°C for 10 min followed by amplification, which was carried out for 25 cycles of denaturation at 94°C for 60 sec, primer annealing at 52°C for 90 sec and extension at 72°C for 90 sec followed by a final extension at 72°C for 15 minutes. Subsequent cloning of the PCR amplified product into the baculovirus transfer vector pAcGP67-A (PharMingen, San Diego, CA, USA) and generation of the recombinant baculovirus expressing the above recombinant protein was performed as described previously [16]. To obtain the recombinant protein, 50 × 10^6 ^*Spodoptera frugiperda *(*Sf*21) insect cells growing in a suspension culture in Spinner bottles (Thermolyne; Barnstead International, Dubuque, IA, USA) were incubated with the recombinant virus at a multiplicity of infection (MOI) of 3 at 42 rotations per minute (rpm) for 96 h after which the cells were pelleted at 1000 g for 15 min. The recombinant protein was purified under denaturing conditions using Ni-NTA resin [25] and subsequently renatured by extensive dialysis against renaturation buffer [50 mM Tris-HCl pH 8.5, 1 mM EDTA (ethylenediaminetetraacetic acid; Amresco, Solon, Ohio, USA), 0.1 mM reduced glutathione (Amresco), 0.01 mM oxidized glutathione (Amresco) and 10% sucrose (Sigma-Aldrich Inc., St. Louis, MO, USA)] for 96 h with 6 changes of the dialysis buffer with decreasing concentrations of urea (4 M, 3 M, 2 M, 1 M, 0.5 M and finally buffer without urea) to assist in removal of urea and refolding of the protein. The refolded protein was further dialyzed against 20 mM Tris pH 7.4. To purify secreted ZP1_273-551aa_, culture supernatant was dialyzed against phosphate buffer (50 mM NaH_2_PO4, 300 mM NaCl, pH 8.0) followed by purification under denaturing conditions using Ni-NTA affinity column essentially as described above from the cell pellet.

### Characterization of recombinant human ZP1_273-551aa_

The purified recombinant human ZP1_273-551aa _was characterized by SDS-PAGE and Western blot as described previously [11]. In brief, the protein (2-5 μg/lane) was boiled for 10 min in 2× sample buffer (0.0625 M Tris pH 6.8, 2% SDS, 10% glycerol, 5% β-mercaptoethanol and 0.01% bromophenol blue) and resolved on a 0.1% SDS-10% PAGE essentially as described previously [11]. For immunoblot, the SDS-PAGE resolved protein was electrophoretically transferred overnight to a 0.45 μm nitrocellulose membrane (Bio-Rad, Hercules, CA, USA) at a constant current of 50 milliampere in Tris-Glycine buffer (25 mM of Tris-HCl and 200 mM glycine) containing 20% methanol. The membrane was then washed once with phosphate buffered saline (PBS; 50 mM phosphate and 150 mM NaCl, pH 7.4) and non-specific sites were blocked with 3% BSA in PBS for 60 min at room temperature (RT). All subsequent incubations were carried out for 1 h at RT and each incubation was followed by three washings with PBS containing 0.1% Tween-20. Post-blocking, the membrane was processed for detection of the recombinant protein by employing 1:100 dilution of mouse polyclonal antibodies against ZP1 synthetic peptide [P5; 26] followed by horse-radish peroxidase (HRP)-conjugated goat anti-mouse immunoglobulin (1:2000; Pierce, Rockford, IL, USA) as secondary antibody essentially as described previously [11]. The blot was developed with 0.6% (w/v) 4-chloro-1-naphthol (Amresco) in 50 mM PBS containing 25% methanol and 0.06% H_2_O_2_.

To determine the glycosylation profile of recombinant human ZP1_273-551aa_, a lectin-binding assay in an Enzyme Linked Immunosorbent Assay (ELISA) format, was performed [11]. As an internal control, *E. coli*-expressed recombinant human ZP1_273-551aa _(unpublished observations) was also used. In brief, microtitration plates (Nunclon™, Rosakilde, Denmark) were coated with the recombinant proteins (500 ng/well) in 50 mM phosphate-buffered saline (PBS), pH 7.4 for 1 h at 37°C followed by overnight incubation at 4°C. All subsequent washings were done three times in 50 mM PBS with 0.05% Tween-20 (PBST). The plate was blocked with 0.1% Tween-20 in PBS (PBST, 200 μl/well) for 90 min at 37°C followed by incubation with 21 biotinylated lectins (1 μg/ml; 100 μl/well) at 37°C for 1 h. The biotinylated lectins available in the Lectin kit-I, II and III (Vector Laboratories, Burlingame, CA, USA) were used in the lectin binding assay. The bound lectins were revealed by incubating with HRP-conjugated streptavidin (1:3000; Pierce; 100 μl/well) at 37°C for 1 h. The enzyme activity was detected by adding 100 μl/well of 0.05% orthophenylenediamine and 0.06% H_2_O_2 _in 50 mM citrate phosphate buffer, pH 5.0 and the reaction was stopped by adding 50 μl/well of 5 N H_2_SO_4_. The absorbance was read at 490 nm with 630 nm as the reference filter.

### Fluorescein isothiocyanate (FITC) labelling of the recombinant proteins

The purified recombinant human ZP1_273-551aa _(1 mg) was dialyzed against 0.5 M carbonate buffer (pH 9.0) and incubated with FITC Isomer I (Pierce) at a molar ratio of 1:24 for 90 min at RT with end-to-end mixing. Post-incubation, unbound FITC was removed from the labelled recombinant protein by extensive dialysis against PBS pH 7.4. All the above treatments were performed under light-protected conditions. The fluorescein/recombinant protein molar ratio (F/P) was determined by spectrophotometric analysis and calculated using the following formula: Molar F/P = (Molecular weight of the protein/389) × [(A_495_/195)/A_280_-{(0.35 × A_495_)}/E^0.1%^]

where: 389 is the molecular weight of FITC in Daltons; 195 is the absorption E^0.1% ^of bound FITC at 495 nm at pH 13.0; (0.35 × A_495_) is the correction factor due to the absorbance of FITC at 280 nm; E^0.1% ^is the absorption at 280 nm of the protein at 1.0 mg/ml

### Binding of recombinant human ZP1_273-551aa _to capacitated human spermatozoa

All experiments using human spermatozoa were carried out under informed consent and following the clearance from the Institutional Bio-safety and Human Ethical Committee. Semen samples were collected from healthy donors and subjected to liquefaction at RT for 30 min. The motile sperm were separated by a two-step Percoll density gradient [27] and processed for capacitation in Biggers-Whitten-Whittingham (BWW) medium [28] supplemented with 2.6% bovine serum albumin (BSA, cell culture grade; Sigma-Aldrich Inc) essentially as described previously [11]. To prepare acrosome-reacted sperm, capacitated sperm were incubated with calcium ionophore A23187 (CaI; 10 μM, Sigma-Aldrich Inc) for 20 min at 37°C and 5% CO_2 _in humidified air. Non-capacitated, capacitated and acrosome-reacted spermatozoa (5 × 10^6^), pre-fixed for 10 min at RT with 0.5% paraformaldehyde, were incubated with 2.5 μg of FITC-labelled recombinant ZP1_273-551aa _in a reaction volume of 50 μl at 37°C and 5% CO_2 _in humidified air for 60 min. In all the experiments, Fetuin (Sigma Aldrich Inc.) labelled with FITC was used as negative control. In addition, FITC-labelled baculovirus-expressed recombinant human ZP1 and ZP3 [12,16] were also used as positive controls. Post-incubation, sperm were processed for simultaneous assessment of the status of acrosome by double labelling with 5 μg/ml tetramethyl rhodamine isothiocyanate conjugated *Pisum sativum* agglutinin (TRITC-PSA; Vector Laboratories Inc., Burlingame, CA, USA) and for binding of FITC-labelled human ZP1_273-551aa _as described earlier [12].

### Induction of acrosome reaction by recombinant human ZP1_273-551aa _in capacitated human spermatozoa

Capacitated sperm (1 × 10^6 ^in BWW medium and 0.3% BSA) were incubated at 37°C with 5% CO_2 _in humidified air for varying times and concentrations of recombinant human ZP1_273-551aa _in a total reaction volume of 100 μl and processed as described earlier [29]. Sperm incubated with BWW supplemented with 0.3% BSA alone accounted for the spontaneous induction of acrosome reaction. Baculovirus-expressed recombinant human ZP1 and ZP3 [[11,16]; 1 μg/reaction] served as positive controls, whereas Fetuin and baculovirus-expressed recombinant human ZP2 [12] served as negative controls in all the experiments. In addition, CaI (10 μM) was also used as positive control in all the experiments. The effect of treatment of various recombinant proteins on sperm with respect to viability by eosin-nigrosin staining [30] and total motility as per the WHO guidelines [31] were assessed. After incubation with various test proteins, sperm were washed twice with 50 mM PBS pH 7.4. Subsequently, sperm pellet was resuspended in 100 μl PBS and 15 μl aliquots were spotted on poly-lysine coated slides (Sigma-Aldrich Inc.) in duplicates, air dried and fixed in chilled methanol for 30 sec. The slides were washed once with PBS and stained with 5 μg/ml TRITC-PSA for 30 min at RT. Sperm showing rhodamine fluorescence in the acrosomal region of the head were classified as acrosome intact while those that demonstrated complete loss of PSA staining in the acrosome or revealed staining at the equatorial region were denoted as acrosome-reacted. All slides were read 'blind' with coded samples. Two hundred sperm were scored for every spot and results are represented as percent stimulation of acrosome reaction which is calculated in the following manner: % stimulation of acrosome reaction = [(AR - negative control)/(CaI - negative control)] × 100

where: AR is the percent induction of acrosome reaction by various inducers, negative control is the spontaneous acrosome reaction and CaI is the percent acrosome reaction by incubation with CaI.

### Elucidation of the downstream signalling events associated with human ZP1_273-551aa _mediated induction of acrosomal exocytosis

Various pharmacological inhibitors at different concentrations, as described previously [16], were employed to delineate the downstream signalling mechanism of the acrosome reaction induced by recombinant human ZP1_273-551aa_. Among these inhibitors are the extracellular calcium chelator: EGTA; L-Type voltage operated Ca^2+ ^channel (VOCC) blockers: Nifedipine & Verapamil; T-Type VOCC blockers: Amiloride & Pimozide; inhibitor of Gi protein: Pertussis toxin (PTX); all of which were pre-incubated with the sperm for 10 min (except PTX, kept for 30 min incubation) at 37°C with 5% CO_2 _in humidified air prior to the addition of ZP1_273-551aa_. All the above inhibitors were procured from Sigma-Aldrich Inc.

### Statistical analysis

In case of binding and induction of acrosome reaction experiments, the results are expressed as mean ± SEM of 3-4 different experiments using semen samples from 3 different male donors. The statistical analysis was done by comparing the means of the negative control (Fetuin) and experimental sets by using one way analysis of variance (ANOVA) followed by Newman-Keuls Multiple Comparison Test. The statistical analysis with respect to the effect of various pharmacological inhibitors on ZP1_273-551aa_-mediated induction of acrosome reaction was performed by Student's t-test. A p value of ≤ 0.05 was considered to be statistically significant.

## Results

### Characteristics of baculovirus-expressed recombinant human ZP1_273-551aa_

**Figure 1:**
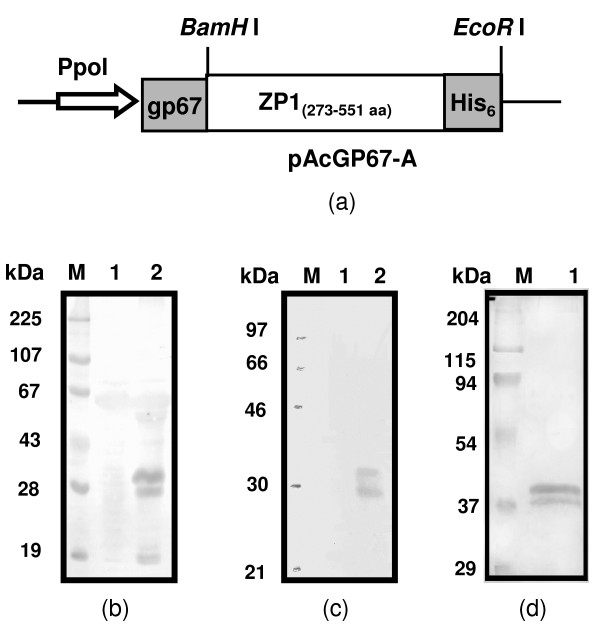
**Design, expression, purification and characterization of the baculovirus-expressed recombinant human ZP1_273-551aa_**. Panel (a) represents the schematic diagram of the cDNA encoding human ZP1_273-551aa _with 6× His tag (His_6_) at C-terminus cloned in baculovirus transfer vector, pAcGP67-A, using *BamH*I and *EcoR*I restriction sites downstream of gp67 insect secretory signal sequence under polyhedrin promoter (Ppol). *Sf*21 cells (0.8 × 10^6^) were infected with the recombinant or wild type AcNPV virus. After 96 h, the infected cells and supernatant were harvested and processed for Western blot as described in *Methods*. Panels (b) & (c) represent immunoblot showing the expression of ZP1_273-551aa _in cell pellet and 10× concentrated culture supernatant respectively from *Sf*21 cell infected with ZP1_273-551aa_-recombinant virus. Lanes 1 and 2 represent the cell pellet or culture supernatant of *Sf*21 cell infected with wild type AcNPV and ZP1_273-551aa_-recombinant virus respectively. Panel (d) represents Coomassie stained SDS-PAGE profile of ZP1_273-551aa _purified from the cell pellet (lane 1, 5 μg/lane). Lane M represents molecular weight markers.

The nucleotide (nt) sequencing of the cDNA encoding ZP1_273-551aa_, PCR amplified from human ovarian cDNA library, revealed three changes at 858, 867 and 1590 nt positions as compared to human ZP1 sequence already published in Genbank (NM_207341). However, these changes did not lead to any change in the deduced aa sequence. Recombinant human ZP1_273-551aa _expressed in *Sf*21 insect cells was present both in the cell lysate as well as the 10× concentrated culture supernatant (Figure 1, panel b and c respectively). The *Sf*21 cells infected with wild type AcNPV, used as negative control, did not show any reactivity with antibodies against ZP1 peptide. The amount of secreted ZP1_273-551aa _present in the culture medium was, however lower as compared to the cell lysate. SDS-PAGE profile of the recombinant human ZP1_273-551aa_, purified using Ni-NTA affinity column from the cell lysate revealed a doublet with molecular weight ranging from ~35-40 kDa (Figure 1, panel d). The recombinant protein purified from the culture supernatant also showed the same profile in Western blot as observed for the cell derived protein (data not shown).

**Figure 2:**
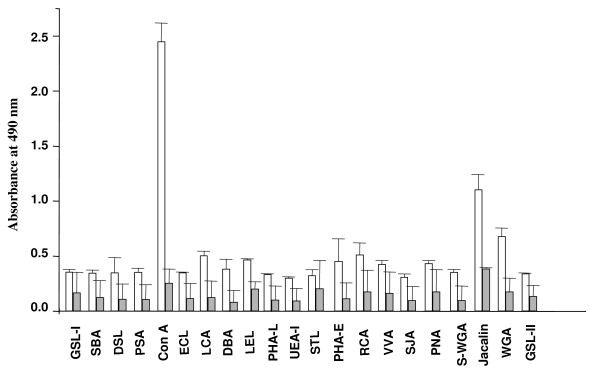
**Profile of lectins binding to baculovirus-expressed human ZP1_273-551aa _in ELISA**. Microtitration plates were coated with the baculovirus-expressed recombinant ZP1_273-551aa _(open bar; 500 ng/well) and processed for evaluation of binding to 21 different biotinylated lectins in an ELISA as described in *Methods*. As an internal control, microtitration plates coated with same amount of *E. coli*-expressed recombinant human ZP1_273-551aa _(grey bar; unpublished observations) were also used. Values are expressed as absorbance obtained with various lectins binding to the respective recombinant protein, after deducting the non-specific binding of the lectins to the uncoated wells. Each bar represents a mean of duplicate experiments and standard deviation of the absorbance values. The lectins tested were GSL-I: *Griffon simplicifolia *lectin I, SBA: Soybean agglutinin, DSL: *Datura stramonium *lectin, PSA: *Pisum sativum *agglutinin, ConA: Concanavalin A, ECL: *Erythrina cristagalli *lectin, LCA: *Lens culinaris *agglutinin, DBA: *Dolichos biflorus *agglutinin, LEL: *Lycopersicon esculentum *lectin, PHA-L: *Phaseolus vulgaris *leucoagglutinin, UEA-I: *Ulex europaeus *agglutinin I, STL: *Solanum tuberosum *lectin, PHA-E: *Phaseolus vulgaris *erythroagglutinin, RCA: *Ricinus communis *agglutinin, VVA: *Vicia villosa *agglutinin, SJA: *Sophora japonica *agglutinin, PNA: Peanut agglutinin, S- WGA: Succinylated Wheat germ agglutinin, Jacalin, WGA: Wheat Germ agglutinin and GSL II: *Griffonia simplicifolia *lectin II.

In lectin binding ELISA, baculovirus-expressed human ZP1_273-551aa _exhibited strong reactivity with Concanavalin A (ConA) and weaker reactivity with Jacalin and Wheat germ agglutinin (WGA) (Figure 2). The *E. coli*-expressed recombinant human ZP1_273-551aa _used as an internal control, failed to show any significant binding to any of the lectins. While ConA has oligosaccharide specificity towards mannose α 1-3 or mannose α 1-6 residues, Jacalin binds to α-O glycosides of Gal or GalNac moieties. WGA has oligosaccharide specificity towards GlcNac and neuraminic acid residues. While ConA and WGA have oligosaccharide specificity towards N-linked sugar residues, Jacalin detects the presence of O-linked carbohydrate moieties.

### Binding characteristics of recombinant human ZP1_273-551aa _with capacitated human spermatozoa

**Figure 3:**
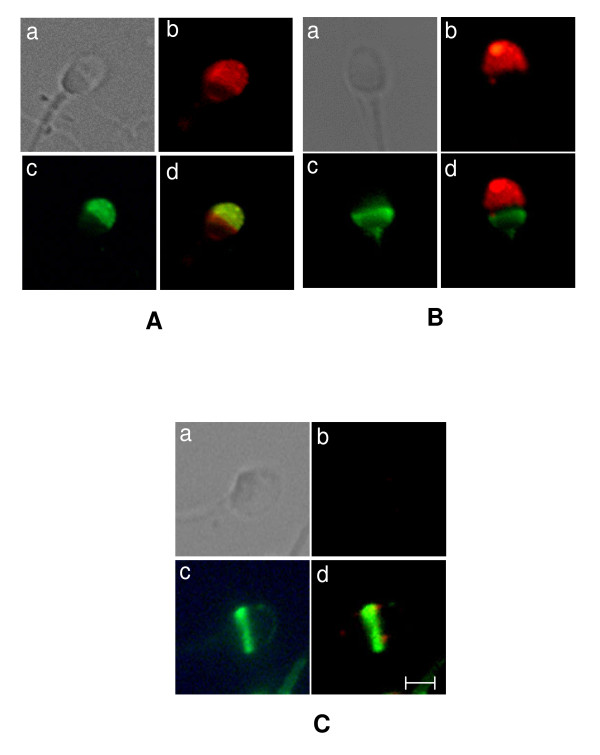
**Binding profile of recombinant human ZP1_273-551aa _with human spermatozoa**. Capacitated/acrosome-reacted sperm (5 × 10^6^/50 μl) were incubated with 2.5 mg of FITC conjugated baculovirus-expressed ZP1_273-551aa _and processed as described in *Methods*. Panels (A) and (B) represent the binding of FITC conjugated ZP1_273-551aa _with capacitated acrosome intact sperm whereas Panel (C) depicts the binding profile with acrosome-reacted spermatozoa. The acrosomal status was determined by labeling the sperm with TRITC-PSA. The images were captured using Eclipse 80*i *fluorescence microscope (Nikon). In each panel, the sub-panels are represented as a: phase contrast; b: TRITC-PSA fluorescence; c: FITC-ZP protein fluorescence and d: overlap of fluorescent frames. The scale bar represents 2.5 μm.

Recombinant ZP1_273-551aa _conjugated to FITC as described in *Methods *revealed molar F/P ratios of 1.1. The FITC-labelled recombinant protein was analyzed for binding to spermatozoa and the acrosomal status of the spermatozoa was simultaneously assessed by double labelling with TRITC-PSA. FITC-labelled recombinant human ZP1_273-551aa _showed very low binding (4.01 ± 1.23%) to non-capacitated human sperm. Hence, all further studies were done using capacitated spermatozoa. The baculovirus-expressed recombinant human ZP1_273-551aa _showed binding to 19.67 ± 2.40% of acrosome-intact and 15.62 ± 1.64% acrosome-reacted sperm (Table 1). Under similar experimental conditions, FITC-conjugated Fetuin, used as a negative control, bound to 3.95 ± 2.12% of acrosome-intact sperm while the positive controls, FITC-labelled baculovirus-expressed ZP3 and full length ZP1 bound to 26.08 ± 2.75% and 15.63 ± 1.88% of the acrosome-intact spermatozoa, respectively (Table 1). Binding analysis with acrosome-reacted sperm revealed that 22.65 ± 2.15% sperm bound to ZP3 and 14.40 ± 1.09% showed binding with full length ZP1 (Table 1). Careful examination of the profile of binding revealed that approximately 70% sperm showed binding of ZP1_273-551aa _to the acrosomal cap (Figure 3A) and approximately 30% in the equatorial region of the acrosome-intact human sperm (Figure 3B). In acrosome-reacted spermatozoa, binding of ZP1_273-551aa _to the acrosomal cap was lost and observed only in the equatorial region (Figure 3C). Further, binding of FITC-labelled baculovirus-expressed ZP3 and full length ZP1 was also observed only in the equatorial segment of the acrosome-reacted spermatozoa [12,16]. In addition, few spermatozoa also showed binding of FITC-labelled recombinant ZP1_273-551aa _to either post acrosomal region (Figure 3B) or mid-piece of acrosome-intact and acrosome-reacted spermatozoa (data not shown).

**Table 1:** Binding characteristics of FITC-labelled recombinant ZP1_273-551aa _with capacitated human spermatozoa in direct binding assay

Recombinant protein	Percent binding to spermatozoa (Mean ± SEM)	Statistical significance^b^
**Acrosome-intact sperm**		
Fetuin	3.95 ± 2.12	
*ZP3	26.08 ± 2.75	p = 0.0004^c^
*ZP1	15.63 ± 1.88	p = 0.0019^c^
ZP1_273-551aa_	19.67 ± 2.40	p = 0.0010^c^
**Acrosome-reacted sperm**		
Fetuin	5.8 ± 3.60	
*ZP3	22.65 ± 2.15^a^	p = 0.0021^c^
*ZP1	14.40 ± 1.09^a^	p = 0.0156^c^
ZP1_273-551aa_	15.62 ± 1.64^a^	p = 0.0117^c^

*Baculovirus-expressed recombinant human ZP1 and ZP3 labelled with FITC;

^a^Binding observed only in the equatorial region

**^b^**Statistical significance with respect to the binding values of Fetuin was calculated by one way ANOVA followed by Newman-Keuls Multiple Comparison Test.

^c^Values are statistically significant.

### The 'ZP domain' of human ZP1 induces acrosomal exocytosis in capacitated human spermatozoa

**Figure 4:**
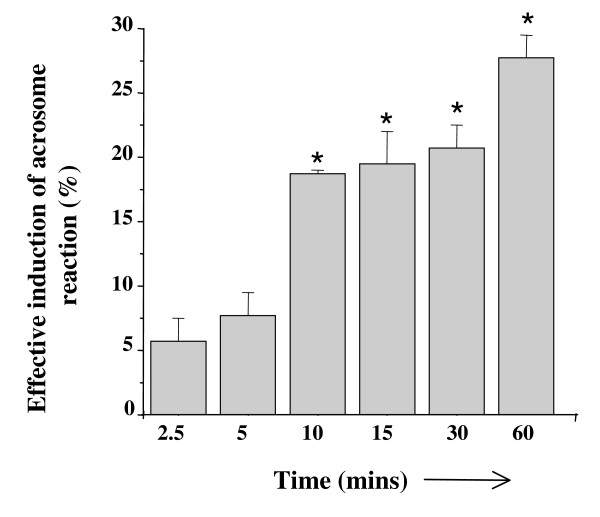
**Time kinetic analysis of ZP1_273-551aa _induced acrosome reaction**. Capacitated sperm (1 × 10^6^/100 μl) were incubated with or without 2 μg/ml of ZP1_273-551aa _for varying time points and subsequently analyzed for acrosomal status by TRITC-PSA staining as described in *Methods*. Y-axis represents effective induction of acrosome reaction which is the percent induction of acrosome reaction in presence of ZP1_273-551aa _minus the percent induction of acrosome reaction observed in the presence of medium alone. Values are Mean ± SEM of 3 different experiments using semen samples from at least two different male donors. *p < 0.01, statistical significance with respect to the effective induction of acrosome reaction mediated by ZP1_273-551aa _at 2.5 minutes.

Recombinant human ZP1_273-551aa _was evaluated for its ability to induce acrosomal exocytosis in capacitated human spermatozoa. No significant decrease in the viability and total motility of the sperm were observed following incubation with the recombinant protein. Capacitated sperm incubated with purified recombinant ZP1_273-551aa _showed a significant (p < 0.05) increase in acrosomal exocytosis as compared to the medium control (Table 2). Baculovirus-expressed recombinant human ZP3, used as a positive control, showed a significant increase in acrosome reaction as compared to Fetuin whereas baculovirus-expressed recombinant human ZP2 [12] failed to do so (Table 2). Dose response studies with baculovirus-expressed human ZP1_273-551aa _revealed a significant increase in the acrosome reaction at 500 ng/ml. The highest acrosome reaction was observed at 2 μg/ml and hence all subsequent experiments were performed using this amount of the recombinant protein. Recombinant ZP1_273-551aa _purified from culture supernatant also exhibited dose dependent induction of acrosomal exocytosis comparable to cell purified recombinant protein (Data not shown). Time kinetic studies revealed that a significant increase in the induction of acrosome reaction could be seen as early as 10 min after exposure of the capacitated sperm to the recombinant ZP1_273-551aa _(Figure 4). Highest level of induction of acrosome reaction was observed at 60 min.

**Table 2:** Induction of acrosome reaction by baculovirus-expressed recombinant human ZP1_273-551aa _in capacitated human spermatozoa

Treatment	Percent stimulation of acrosome reaction^a^ (Mean ± SEM)	Statistical significance^b^
Fetuin (10 μg/ml)	7.6 ± 1.8	
ZP1 (10 μg/ml)	33.2 ± 2.9	p = 0.0065*
ZP2 (10 μg/ml)	10.9 ± 2.4	p = 0.6085
ZP3 (10 μg/ml)	42.3 ± 3.2	p = 0.0023*
ZP1_273-551aa_		
500 ng/ml	17.2 ± 1.6	p = 0.0391*
1 μg/ml	30.1 ± 0.6	p = 0.0078*
2 μg/ml	58.2 ± 1.7	p = 0.0002*
5 μg/ml	40.5 ± 0.6	p = 0.0007*
10 μg/ml	41.6 ± 1.7	p = 0.0016 *

**^a^**Percent stimulation of acrosomal exocytosis was calculated as stated in Methods. Values represent mean ± SEM of at least four independent experiments.

**^b^**Statistical significance with respect to the acrosome reaction induced by Fetuin was calculated by one way ANOVA followed by Newman-Keuls Multiple Comparison Test.

*Values are statistically significant.

### Downstream signalling events associated with ZP1_273-551aa_-mediated induction of acrosomal exocytosis

**Figure 5:**
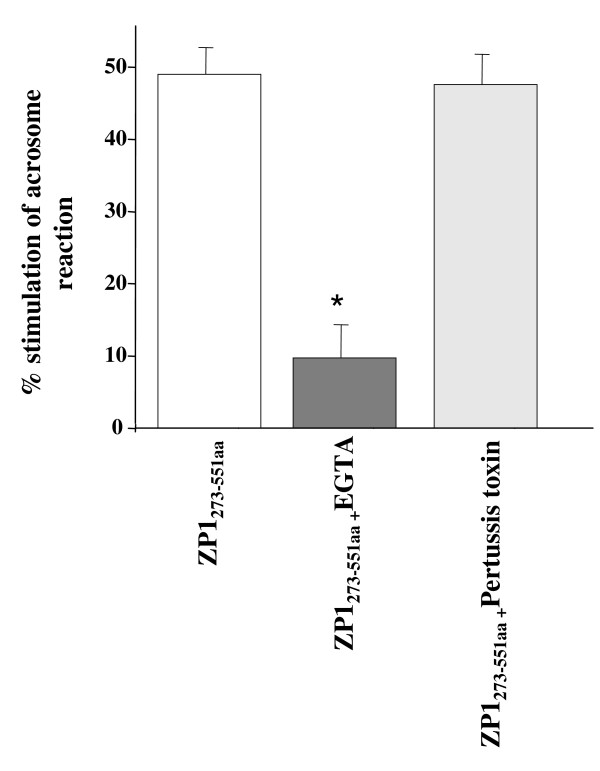
**Role of extracellular calcium and G_i _protein-coupled receptor in ZP1_273-551aa_- mediated induction of acrosomal exocytosis**. Capacitated sperm (1 × 10^6^/100 μl) were pre-treated with 8 mM EGTA (10 min prior-incubation) and 0.1 μg/ml PTX (30 min prior-incubation) to study effects of extracellular calcium and G_i _protein respectively. These pretreated sperm were incubated with ZP1_273-551aa _(2 μg/ml) for 60 min, and subsequently analyzed for acrosomal status by TRITC-PSA staining as described in *Methods*. Spontaneous acrosome reaction was assessed by incubating sperm only with BWW medium. Data has been represented as percent stimulation of acrosome reaction calculated as described in *Methods*. Values are Mean ± SEM of 3 different experiments using semen samples from at least two different male donors. *p < 0.01, statistical significance with respect to the percent stimulation of acrosome reaction mediated by ZP1_273-551aa_.

**Figure 6:**
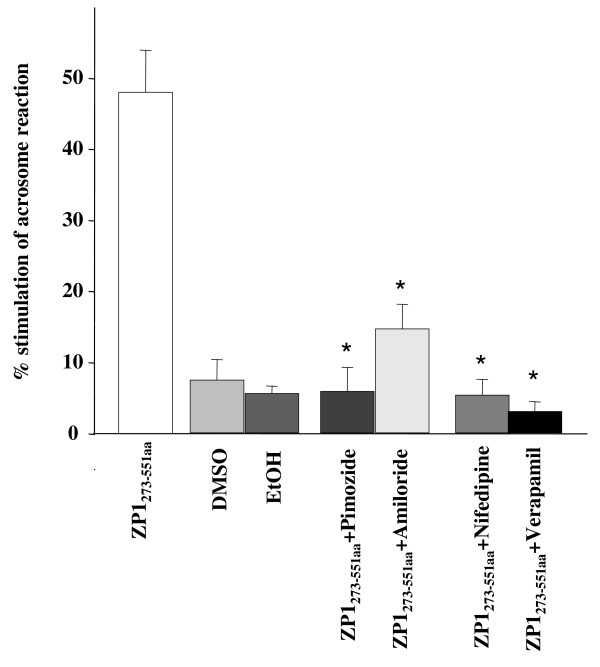
**Role of voltage operated Ca^2+ ^channels (VOCCs) in ZP1_273-551aa_- mediated induction of acrosomal exocytosis**. Capacitated sperm (1 × 10^6^/100 μl) pre-treated with 10 μM Verapamil, 10 μM of Nifedipine (10 min prior-incubation with each to study effect of blocking of L-type VOCC), 10 μM Pimozide and 100 μM Amiloride (10 min pre-incubation; to study effect of blocking of T-type VOCC) were incubated with 2 μg/ml of ZP1_273-551a _for 60 min and subsequently analyzed for acrosomal status by TRITC-PSA staining as described in *Methods*. Spontaneous acrosome reaction (spontaneous AR) was analyzed by incubating sperm only with BWW medium. Additional negative controls comprised of the same amount of dimethyl sulphoxide (DMSO) and ethanol (EtOH) as used to dissolve T- and L-type VOCC inhibitors. Data has been represented as percent stimulation of acrosome reaction calculated as described in *Methods*. Values are Mean ± SEM of 3 different experiments using semen samples from at least two different male donors. *p < 0.05, statistical significance with respect to the percent stimulation of acrosome reaction mediated by ZP1_273-551aa_.

To elucidate the downstream signalling pathway involved in acrosomal exocytosis mediated by the 'ZP domain' of ZP1, different pharmacological inhibitors were employed. The average viability in absence of pharmacological inhibitors of the capacitated sperm ranged from 85-90%. No significant change in the sperm viability was observed subsequent to incubation with various pharmacological inhibitors. No significant decrease in ZP1_273-551aa _mediated induction of acrosome reaction was observed (p > 0.05) after incubation with Pertussis toxin (0.1 μg/ml; 30 min pre-incubation) pre-treated capacitated human spermatozoa (Figure 5) suggesting that activation of Gi-protein coupled receptor is not necessary for 'ZP domain' of ZP1 mediated acrosomal exocytosis. Induction of acrosomal exocytosis mediated by ZP1_273-551aa _required extracellular Ca^2+ ^as prior incubation of capacitated sperm in BWW medium with EGTA before exposing to recombinant protein led to a significant decrease in acrosomal exocytosis (p < 0.01, Figure 5). Both L- and T- type Ca^2+ ^channels seem to play a role in ZP1_273-551aa _mediated induction of acrosome reaction as inhibitors for both types of channels resulted in a significant decrease in acrosomal exocytosis (Figure 6).

## Discussion

In order to understand the molecular basis of fertilization in humans, it is imperative to delineate the role of ZP glycoproteins during different stages of fertilization. In this direction, a recent study demonstrated that human ZP1 in addition to ZP3 and ZP4 binds to capacitated human spermatozoa and induces acrosomal exocytosis [16]. To investigate whether the 'ZP domain' of human ZP1 also plays a functional role, the fragment 273-551 aa residues, comprising of the 'ZP domain' of ZP1 was cloned and expressed in the baculovirus expression system. We have opted for the baculovirus expression system over the mammalian expression system as recombinant human ZP1, ZP3 and ZP4 obtained using this expression system not only bind to capacitated acrosome-intact spermatozoa but also induce acrosome reaction in a dose dependent manner [9,11,12,16,32]. These studies are further corroborated by observations that baculovirus-expressed recombinant rabbit 55 kDa protein (homolog of human ZP4) binds in a dose dependent manner to the rabbit sperm [33]. However, recombinant porcine ZP4 expressed in insect cells binds to bovine but not porcine sperm which may be due to the glycosylation profile of recombinant protein being similar to bovine rather than porcine ZP4 [34]. Transfer vector (pAcGP67-A) having the gp67 insect signal sequence was used to express human ZP1_273-551aa_, which facilitates its proper post-translational processing through ER-Golgi pathway. Further, the levels of expressed protein are high as an average yield of 250-500 μg of purified baculovirus-expressed protein was obtained from each round of purification using 150 × 10^6 ^recombinant virus-infected *Sf*21 cells, thus facilitating availability of sufficient amounts of purified protein to perform various assays.

The baculovirus-expressed human ZP1_273-551aa _showed two bands in SDS-PAGE as well as Western blot which may be due to differential glycosylation of the expressed protein. Expression of human ZP1_273-551aa _in *Sf*21 cells in presence of Tunicamycin (20 μg/ml, Sigma-Aldrich Inc), which inhibit N-linked glycosylation, resulted in a single band (data not shown) suggesting that the two bands represent different glycoforms of expressed recombinant protein. Observation of more than one band of human zona glycoproteins expressed using baculovirus expression system has also been reported previously [29]. The apparent higher molecular weight of the baculovirus-expressed protein as compared to the calculated molecular weight of 30.8 kDa may be due to the glycosylation as revealed by the binding of various lectins. Binding of recombinant human ZP1_273-551aa _with ConA (specific for mannose α 1-3/1-6 residues, N-linked), WGA (GlcNac and neuraminic acid residues, N-linked) and Jacalin (specific for α-O glycosides of Gal or GalNAc moieties, O-linked) suggest the presence of both N- and O-linked glycosylation. Using immunocytochemistry, a study has also shown the presence of both ConA and Jacalin binding to the native human ZP [35]. The presence of very high concentration of D-mannose residues in human ZP has earlier also been documented, reflecting a high content of asparagine-linked oligosaccharides [36]. Characterization of the glycosylation profile of the purified native human ZP3 and ZP4 revealed that it is predominantly N-linked [13].

It was observed that recombinant baculovirus-expressed ZP1_273-551aa _was secreted in the supernatant in very low amounts. It has been reported previously that the secretion of a lutropin protein receptor expressed using the baculovirus expression system was dependant both on the presence of the signal peptide as well as the promoter [37]. The temporal activity (early or late) of a promoter induces drastic changes in the pattern of protein processing. In *Sf9 *insect cells, the secretory pathway of the cells was seen to be compromised in the late stages of baculovirus infection [38,39]. Using the late core-protein promoter instead of the very late polyhedrin promoter, secretion of beta-human chorionic gonadotropin was increased, although not at the level of the native protein [40]. Hence, expression of recombinant human ZP1_273-551aa _under the control of very late polyhedrin promoter may be one of the reasons behind the poor levels of secretion of the recombinant protein in spite of the presence of insect secretory sequence in the pAcGP67-A vector. Hence, functional studies were performed using the protein purified from the cell pellet and subsequently induction of acrosome reaction was confirmed with the protein purified from the culture supernatant.

Studies have shown that in humans ZP1, ZP3 and ZP4 bind to capacitated human sperm [12,13,16]. In the present study, for the first time, it has been demonstrated that the 'ZP domain' module of ZP1 binds to capacitated spermatozoa. The observed higher bindings (statistically non significant) of 'ZP domain' of ZP1 as compared to full length ZP1 to the capacitated acrosome-intact spermatozoa may be due to the differences in the accessibility of the binding domains/regions present on these proteins to the spermatozoa. Low binding percentage of 'ZP domain' of ZP1 to capacitated acrosome-intact spermatozoa in the present study may be corroborated by a report where more than 75% of motile sperm from fertile men have been shown to be incapable of binding to native ZP [41]. An alternate plausible explanation for low binding may also be due to different maturation stages of the sperm present in the human semen. The observed low binding may not be due to the impaired biological activity of recombinant human ZP1_273-55aa _post-labelling with FITC as comparable binding percentages were observed when either FITC-labelled baculovirus-expressed recombinant human ZP3 and ZP4 or solubilized human ZP were used as shown previously [12].

Successful mammalian fertilization requires capacitated spermatozoa to undergo acrosome reaction. High resolution scanning electron microscopy studies revealed that the ZP matrix of human oocytes is made-up of a delicate meshwork of thin interconnected filaments in a regular alternating pattern of wide and tight meshes/pores [42]. Capacitated human sperm incubated with intact human zonae or acid disaggregated zonae led to a significant increase in acrosome reaction [43,44]. To delineate the role of individual zona proteins in the induction of acrosome reaction, various groups have either used the purified protein from native source (difficult to rule out minor contaminants of other egg associated or zona proteins) or recombinant protein. The studies presented in this manuscript showed that the baculovirus-expressed recombinant human ZP1_273-551aa _was able to induce acrosome reaction. The dose-response results indicate that as little as 500 ng/ml of baculovirus-expressed recombinant ZP1_273-551aa _is sufficient to induce a significant acrosome reaction in capacitated human sperm, though the maximum induction of acrosome reaction was observed at 2 μg/ml (Table 2). The amount of recombinant protein required to induce acrosome reaction far exceeds that present in the ZP matrix. It may be due to the presence of other factors in the female reproductive tract that may act in synergy with the zona proteins to bring about acrosomal exocytosis in the sperm [45]. Further, based on recent studies by our group and others, the observed acrosomal exocytosis mediated by ZP may be due to a combined effect of ZP1, ZP3 and ZP4 [9,11,12,14,16,32]. The observed ability of the baculovirus-expressed ZP1_273-551aa _to induce acrosome reaction is not due to post mortem acrosomal loss as no change either in the sperm percent motility or in the sperm viability was observed when the sperm were incubated with the recombinant protein.

It has been proposed that ZP glycoproteins mediate acrosomal exocytosis involving two different signalling pathways. One is a tyrosine kinase receptor coupled to phospholipase Cγ (PLCγ) and the other is the G_i _protein-coupled receptor that activates phospholipase Cβ_1 _(PLCβ_1_) mediated signalling pathway [14,46,47]. Pertussis toxin, an inhibitor of G_i _protein mediated signalling pathway did not inhibit the acrosome reaction mediated by recombinant baculovirus-expressed human ZP1_273-551aa _suggesting that it acts in a similar fashion as human ZP4 and the full length human ZP1 [11,14,16]. However, the same concentration of Pertussis toxin under similar experimental conditions significantly inhibited the baculovirus-expressed human ZP3 mediated induction of acrosome reaction [11]. These results suggest that ZP3 uses G_i _protein-coupled receptor pathway whereas ZP1/ZP4 are not dependent on its activation to induce acrosomal exocytosis.

Extracellular Ca^2+ ^is required to bring about ZP1- mediated acrosomal exocytosis [16] and both T- and L-type VOCCs are involved in the downstream signalling pathway. The concentrations of the VOCCs inhibitors employed in these studies were decided upon by reviewing the various studies done employing the same inhibitors, published previously [14,48,49]. The above studies therefore, reveal that the downstream signalling pathway of ZP1_273-551aa_-induced acrosome reaction shows similarity to the one elucidated for full length human ZP1 and ZP4 [14,16]. Both follow the G_i _protein independent pathway, both involve activation of L- and T- type VOCCs [14,16]. These findings can be vindicated by the fact that within the four human ZP glycoproteins, ZP1 and ZP4 share the maximum sequence identity of 47% with each other hence, the similarity in their mechanism of action.

All ZP glycoproteins (except for cat ZP3 and mouse ZP1) share a homologous region designated as the 'ZP domain' which is also present in several eukaryotic extra-cellular proteins [19,21]. The 'ZP domain' consists of approximately 260 aa including 8 conserved Cys residues and is predicted to have high β-strand content with additional conservation of hydrophobicity, polarity and turn forming tendency at a number of positions [19,21]. In humans, 'ZP domain' module of ZP1 corresponds to 279-549 aa, ZP2 from 372 to 637 aa, ZP3 from 45 to 303 aa and ZP4 from 188-460 aa. Comparison of the aa sequence of 'ZP domain' of human ZP1 revealed sequence identity of 29% with the 'ZP domain' of ZP2, 18% with ZP3 and 47% with ZP4, suggesting thereby significant variations at the aa level in spite of having conserved 'ZP domain' motif. Human ZP3 'ZP domain' consists of two conserved sub-domains, N-terminal (ZP-N) followed by internal hydrophobic patch (IHP, 167-173 aa) and C-terminal (ZP-C) followed by external hydrophobic patch (EHP, 362-368 aa) separated by a short protease sensitive hinge. The polymerisation property of the 'ZP domain' is most likely imparted by the presence of the ZP-N sub domain as PLAC-1 like proteins consisting of only ZP-N domain can polymerize and majority of 'ZP domain' mutations causing disease in humans, such as those in α-tectorin and Tamm-horsfall protein, are clustered within the first half of the domain [20,23,50-52]. ZP-C sub-domain on the other hand, is found only as part of a complete 'ZP domain' and can adopt different disulfide connectivities [21,53,54] and hence, it may play a crucial role in regulating the specificity of the ZP-N sub-domain to determine whether or not a given 'ZP domain' protein can homo- or hetero-polymerize. Using various fragments of baculovirus-expressed recombinant human ZP3, we showed that the fragments corresponding to 214-348 aa and 214-305 aa are able to induce AR where as fragment corresponding to 1-175 aa failed to do so [29]. These experiments suggest that the C-terminal fragment of ZP3 'ZP domain' is involved in the induction of AR. Further studies are required to delineate the role of 'ZP domain' of ZP2 and ZP4 in induction of AR. How crucial is the glycosylation pattern to determine 'ZP domains' ability of different zona proteins to induce AR needs to be investigated. The unpublished observations from our group has shown that *E. coli*-expressed 'ZP domain' of human ZP1, presumably devoid of glycosylation, failed to induce AR suggesting that glycosylation have critical role in induction of AR.

In humans, in addition to ZP3, ZP1 and ZP4 also induce acrosome reaction [5-9,11,12,14,16]. Studies from various other species such as chicken, pig, rabbit and bonnet monkey have also suggested that more than one zona protein is involved in binding to the capacitated spermatozoa and induction of acrosome reaction [33,55-57]. Delineation of downstream signalling pathway revealed that human ZP3 involves G_i _protein-receptor coupled pathway and primarily use T-type VOCCs whereas induction of AR by human ZP1/ZP4 is independent of G_i _protein-receptor coupled pathway and involve both L- and T- type VOCCs. The results presented in this manuscript suggest, for the first time, that the 'ZP domain' of recombinant human ZP1 has functional activity.

## Competing interests

The authors declare that they have no competing interests.

## Authors' contributions

AG and SKG participated in the study design, execution, analysis and manuscript writing. TG helped in purification of recombinant protein and PB performed additional experiments using recombinant protein purified from the culture supernatant. All the authors have read and approved the final manuscript.
